# Stereotactic body radiotherapy for stage I lung cancer and small lung metastasis: evaluation of an immobilization system for suppression of respiratory tumor movement and preliminary results

**DOI:** 10.1186/1748-717X-4-15

**Published:** 2009-05-28

**Authors:** Fumiya Baba, Yuta Shibamoto, Natsuo Tomita, Chisa Ikeya-Hashizume, Kyota Oda, Shiho Ayakawa, Hiroyuki Ogino, Chikao Sugie

**Affiliations:** 1 Department of Radiology, Nagoya City University Graduate School of Medical Sciences, Nagoya, Japan; 2 Department of Radiation Oncology, Aichi Cancer Center Hospital, Nagoya, Japan; 3 Nagoya Radiosurgery Center, Nagoya Kyoritsu Hospital, Nagoya, Japan; 4 Department of Radiation Therapy, Aizawa Hospital, Matsumoto, Japan

## Abstract

**Background:**

In stereotactic body radiotherapy (SBRT) for lung tumors, reducing tumor movement is necessary. In this study, we evaluated changes in tumor movement and percutaneous oxygen saturation (SpO_2_) levels, and preliminary clinical results of SBRT using the BodyFIX immobilization system.

**Methods:**

Between 2004 and 2006, 53 consecutive patients were treated for 55 lesions; 42 were stage I non-small cell lung cancer (NSCLC), 10 were metastatic lung cancers, and 3 were local recurrences of NSCLC. Tumor movement was measured with fluoroscopy under breath holding, free breathing on a couch, and free breathing in the BodyFIX system. SpO_2 _levels were measured with a finger pulseoximeter under each condition. The delivered dose was 44, 48 or 52 Gy, depending on tumor diameter, in 4 fractions over 10 or 11 days.

**Results:**

By using the BodyFIX system, respiratory tumor movements were significantly reduced compared with the free-breathing condition in both craniocaudal and lateral directions, although the amplitude of reduction in the craniocaudal direction was 3 mm or more in only 27% of the patients. The average SpO_2 _did not decrease by using the system. At 3 years, the local control rate was 80% for all lesions. Overall survival was 76%, cause-specific survival was 92%, and local progression-free survival was 76% at 3 years in primary NSCLC patients. Grade 2 radiation pneumonitis developed in 7 patients.

**Conclusion:**

Respiratory tumor movement was modestly suppressed by the BodyFIX system, while the SpO_2 _level did not decrease. It was considered a simple and effective method for SBRT of lung tumors. Preliminary results were encouraging.

## Background

Stereotactic body radiotherapy (SBRT) is now spreading worldwide as a new treatment modality for stage I non-small cell lung cancer (NSCLC). Following the pioneering work by Uematsu et al. [1,2], promising clinical results with excellent local control and low complication rates have been reported. Clinical outcomes on 257 patients from 14 institutions in Japan were published recently, which showed a 5-year survival rate of 71% in medically operable patients receiving sufficient radiation doses [3]. At present, SBRT is considered a therapeutic option in stage I NSCLC either for inoperable patients or for patients refusing surgery. SBRT for lung cancer is under evaluation in clinical trials. Japan Clinical Oncology Group (JCOG) conducted a phase II study 0403 of SBRT in operable and medically inoperable patients with pathologically proven T1N0M0 NSCLC to evaluate efficacy and safety. JCOG 0702, a phase I dose escalation study of SBRT in patients medically inoperable or unfit for surgery with pathologically proven T2N0M0 NSCLC, has started to determine the recommended dose. Radiation Therapy Oncology Group (RTOG) is developing a phase II trial 0236 and 0618 of SBRT. These trials are designed for patients with pathologically proven, inoperable and operable T1, T2, T3 (chest wall primary tumors only), N0, M0 NSCLC. The primary endpoint is 2-year local control. Results of these studies are awaited.

A lung tumor is a movable target so that management of tumor motion is required for SBRT of lung tumors. The lung tumor movement can result from respiration, cardiac motion and aortic pulsation. While it is difficult to diminish the non-respiratory organ motion, there are some approaches to reduce the respiratory organ motion [4-6]. Accurate set-up is required for SBRT, so immobilization devices are used for diminishing the positioning error, i.e. repositioning accurately. Some of them also have effect of diminishing the organ motion errors, i.e. reducing the tumor movement. Among several devices that have been developed for immobilization, we have used the BodyFIX system (Medical Intelligence, Schwabmuenchen, Germany) [7]. It is one of commercially available immobilization devices, and is designed to readily fix patients body and to suppress respiratory movement. In this study, we measured motion of lung tumors, and examined suppression of respiratory tumor movement when using the BodyFIX system. We also monitored the percutaneous oxygen saturation (SpO_2_) level with a finger pulseoximeter while using the BodyFIX system. In addition, we report clinical outcomes of SBRT for lung tumors performed with this immobilization system.

## Methods

### Patient Characteristics

Between February 2004 and June 2006, 53 patients underwent stereotactic body radiotherapy (SBRT) for a lung tumor. Two patients received SBRT twice for different lesions, so a total of 55 lesions were treated. Accordingly, lung tumor movement and changes of SpO_2 _levels were measured 55 times. There were 39 men and 14 women. The age at SBRT ranged from 16 to 86 years, with a median of 74 years. The eligibility criteria for the patients were as follows: (1) histologically-confirmed primary NSCLC diagnosed as T1N0M0 or T2N0M0 stage according to the International Union Against Cancer (UICC) 1997 system by CT scans, bone scintigraphy and brain magnetic resonance imaging (MRI), or clinically diagnosed as recurrent or metastatic lung cancer; (2) tumor diameter ≤ 50 mm, and (3) World Health Organization performance status ≤ 2. When 18-fluoro-deoxyglucose-positron emission tomography (FDG-PET) was performed, bone scintigraphy was omitted. FDG-PET was performed in 24 patients with primary NSCLC, 2 with lung metastasis, and 2 with postoperative local recurrence of NSCLC. Although the diagnosis of primary NSCLC could not be confirmed with CT-guided biopsy in 1 patient, this case was included in the study considering the positive FDG-PET finding and the increase in tumor size during observation period. Of the 55 lesions, 42 were primary NSCLC, 10 were metastatic lung cancer, and 3 were postoperative local recurrence of NSCLC. The tumor diameter ranged from 10 to 50 mm with an average of 26 mm. Of the 55 lesions, tumor location was the right upper, middle and lower lobes in 17, 2 and 13 cases, respectively, and the left upper and lower lobes in 13 and 10 cases, respectively. All of them were treated using the BodyFIX immobilization device. Patient characteristics are summarized in Table 1. In all patients, pulmonary functions were assessed before SBRT. Respiratory functions were categorized as obstructive dysfunction when the ratio of forced expiratory volume in 1 second to forced vital capacity (FEV 1.0%) was less than 70%, as constrictive dysfunction when the percent vital capacity (%VC) was less than 80%, and as mixed dysfunction when both criteria were fulfilled.

**Table 1:** Patient characteristics

Patients/Lesions		53/55
Male/Female		39/14 (41/14)*
Age (years)	median (range)	74 (16–86)
Tumor size (mm)	average (range)	26 (10–50)
Disease	Primary lung cancer	42
	Stage IA/IB	30/12
	Operable/Inoperable	7/35
	Sq/Ad/NSC/Unspecified**	14/22/5/1
	Local recurrence	3
	Metastatic lung cancer	10
Follow-up period (month)		32 (24–52)

*for all 55 lesions treated.

**Sq = squamous cell carcinoma; Ad = Adenocarcinoma; NSC = Non-small-cell carcinoma.

### Immobilization System

**Figure 1:**
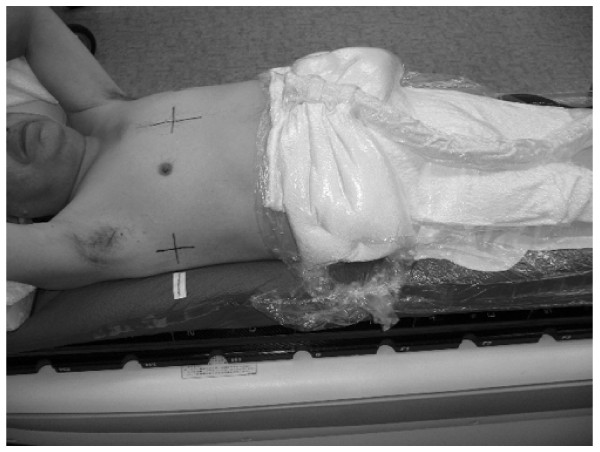
**The BodyFix immobilization system**. Patient lied in the molded vacuum cushion with a T-shaped holding bar, the marks on the patient's skin and the vacuum pillow being matched. The air among the clear plastic sheet, patient, and the vacuum cushion was evacuated covering the patient's lower body up to the abdomen with the clear plastic sheet.

The BodyFIX system consists of a vacuum cushion, a clear plastic sheet covering the patient's torso and lower extremities, and a vacuum pump. The patient lied in supine position on a vacuum cushion. Both arms were raised using a T-shaped holding bar. The vacuum cushion was filled with small styrofoam balls, and the enclosed air was evacuated through a vacuum pump, so that the cushion was molded to the patient's posterior body surface. The reference marks were drawn on the patient's skin and the vacuum cushion to locate the patient body in the same position repeatedly. A clear plastic sheet was attached to the lower sides of the vacuum cushion covering the patient's lower body up to the abdomen or thorax. The air among the clear plastic sheet, patient, and vacuum cushion was evacuated with a pressure of 80 mbar, while the vacuum cushion retained its mold. Once the vacuum cushion was molded to the patient's back and sides, the air among the clear plastic sheet, patient, and the vacuum cushion was evacuated; then the system became a rigid immobilization device (Figure 1), and patients underwent treatment planning CT and SBRT under these conditions.

### Methods for Suppression of Respiratory Tumor Movement

To investigate the optimal methods for suppression of respiratory tumor movement using the BodyFIX system, motion of tumor was measured with fluoroscopy under the following conditions; A: breath holding, B: free breathing on a couch, C: free breathing in the BodyFIX system with a patient's lower body covered up to the upper abdomen with a clear plastic sheet, D: free breathing in the BodyFIX with a patient's lower body covered up to the upper thorax with a clear plastic sheet. Fluoroscopy measurements were performed prior to CT scan for treatment planning. Patients were positioned in the molded vacuum cushion on the couch of an X-ray simulator to estimate tumor movement. A cross-line scale was superimposed on the screen of fluoroscopy to measure the amplitude of tumor movement. The amplitude was measured visually by at least 3 staff members in the anteroposterior (AP) and lateral directions for 30 seconds each after respiration became stable. Measurements were possible for all tumors in both directions.

### Percutaneous Oxygen Saturation

To evaluate whether or not the immobilization methods affected the oxygenation status, SpO_2 _levels were measured with a finger pulseoximeter every 15 seconds for 2 minutes under conditions B, C and D. For control, SpO_2 _was also measured under breath holding (condition A) every 5 seconds for up to 40 seconds.

### Treatment Planning

CT images for treatment planning were acquired using a CT simulator (Mx8000, Philips Medical Systems, Best, the Netherlands) after patients were positioned in the BodyFIX system in the supine position. First CT scan was performed under normal breathing. Then 2 additional scans were performed with breath holding at the expiratory and inspiratory phases. The table pitch was 0.75 mm/second, and the rotation time was 2 seconds under the free-breathing conditions and 1 second under the expiratory and inspiratory breath-holding conditions. All CT scans were reconstructed at 2.5 mm thickness.

The outlines of the target were delineated on a 3-dimensional radiation treatment planning system (3D RTPS) (Eclipse Version 7.5.14.3, Varian Medical Systems, Palo Alto, California, USA) using lung CT window setting (window width: 1300 Hounsfield units (HU); and window level: -350 HU, typically). The clinical target volume (CTV) was defined by the visible gross tumor volume (GTV). The CTV on CT at the 3 phases were superimposed on the 3D RTPS to represent the internal target volume (ITV).

CT was taken just before the first and third fractions of SBRT to evaluate the accuracy of reproducibility of patient and tumor position, and any positioning error was corrected; patients were then transferred to the treatment couch together with the BodyFix system. In addition, AP and lateral portal images were obtained for verification before every treatment. They were compared visually with digitally reconstructed radiograph (DRR) derived from the planning CT scan by at least 3 staff members in relation to bony structures. We measured setup errors for 40 times in the first 10 patients. Absolute setup errors were ≤ 5 mm in 93%, 90% and 78%, and ≤ 10 mm in 100%, 100% and 98% in lateral (right-to-left, RL), AP and craniocaudal (CC) directions, respectively. So we defined the planning target volume (PTV) margin for the ITV to be 5 mm in the RL and AP directions, and 10 mm in the CC direction. The patient was repositioned if the setup error was greater than 3 mm in any direction.

Three coplanar and 4 noncoplanar static ports were used. The beam arrangement was selected for the gantry not to collide with the patient and the BodyFix system. We avoided the interference of the thick carbon bars that lie on the right and left sides of the couch. Dry run was performed to choose appropriate beam arrangement before SBRT was performed.

SBRT was delivered by a linear accelerator (CLINAC 23EX, Varian Medical Systems, Palo Alto, California, USA) with 6-MV photons. The planned dose was 44 Gy in 4 fractions for tumors with a maximum diameter of less than 1.5 cm, 48 Gy in 4 fractions for tumors with a maximum diameter of 1.5–3 cm, and 52 Gy in 4 fractions for those larger than 3 cm. A total dose of 34 or 36 Gy in 2 fractions was delivered for metastatic lung cancers with a maximum diameter of less than 1.5 cm. Pencil beam convolution with Batho power law correction of the Eclipse system was used for dose calculation algorithm. The dose was prescribed at the isocenter; 95% of the PTV was ensured to be covered with at least 80% of the prescribed isocenter dose. Since the total irradiation time was less than 30 minutes per fraction, intrafractional tumor movement was not measured.

### Statistical Analysis

Paired *t*-test was used to examine differences in tumor movement between different patient conditions. A correlation coefficient was calculated to assess the relationship between the respiratory function and tumor movement. To compare the change of SpO_2 _levels under conditions C and D, repeated measure analysis of variance was used. Survival rates and cumulative incidences of complications were calculated by the Kaplan-Meier method.

## Results

### Respiratory Tumor Movement

**Figure 2:**
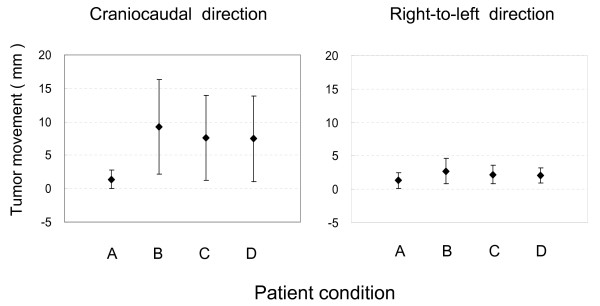
**Amplitude of tumor movement under conditions A, B, C and D in the craniocaudal and right-to-left directions**. A: breath holding; B: free breathing; C: free breathing covering the patient's lower body up to the abdomen with a clear plastic sheet; D: free breathing covering the patient's lower body up to the thorax with a clear plastic sheet. Bars represent SD.

Patient compliance with the BodyFIX system was 100%. Amplitude of tumor movement is shown in Table 2 and Figure 2. Under breath-holding conditions, the average tumor movement was less than 2 mm in both CC and RL directions, whereas it was 7–10 mm in the CC direction and 2–3 mm in the RL direction under the other three conditions. Statistical differences in tumor movement among conditions B, C and D are shown in Table 3. By covering the patient's lower body up to the upper abdomen or upper thorax with the sheet (condition C or D), respiratory tumor movements were slightly but significantly reduced in both CC and RL directions, compared with free-breathing condition B. There were no differences in tumor movement between conditions C and D.

**Table 2:** Amplitude of tumor movement (mm)

	Craniocaudal direction (n = 55)				Right-to-left direction (n = 55)			
	A	B	C	D	A	B	C	D
		
Range	0 – 5	1 – 25	0 – 24	0 – 25	0 – 5	0 – 10	0 – 5	0 – 5
Mean ± SD	1.4 ± 1.4	9.2 ± 7.1	7.6 ± 6.4	7.5 ± 6.4	1.3 ± 1.1	2.7 ± 1.9	2.2 ± 1.4	2.1 ± 1.2

A: Breath holding.

B: Free breathing on a couch.

C: Free breathing covering the patient's lower body up to the abdomen with a clear plastic sheet.

D: Free breathing covering the patient's lower body up to the thorax with a clear plastic sheet.

**Table 3:** Reduction of tumor movement

	Craniocaudal direction			Right-to-left direction		
	B – C†	B – D†	C – D†	B – C†	B – D†	C – D†
		
≤ - 3 mm	2	2	4	0	0	0
- 2 ≤, ≤ 2 mm	38	38	47	51	49	55
≥ 3 mm	15	15	4	4	6	0
p*	0.0006	0.0001	0.50	0.0009	0.0003	0.15

p*: Paired *t*-test for mean values of tumor movement.

X – Y†: Amplitude of tumor movement in condition X minus that in condition Y.

The tumor movement was defined as increased and decreased when the amplitude in condition B minus the amplitude in condition C or D exceeded - 2 and 2 mm, respectively; otherwise, the tumor movement was regarded as no change. Under both conditions C and D, the tumor movement increased in 2 cases, did not change in 38 cases, and decreased in 15 (27%) cases, compared with the free-breathing condition B in the CC direction. In the RL direction, the tumor movement did not change in 51 (93%) and 49 (89%) cases and decreased in 4 (7%) and 6 (11%) cases under conditions C and D, respectively. Thus, respiratory tumor movement in the CC direction was reduced by 3 mm or more in about one-quarter of the patients by covering the patient's lower body up to the upper abdomen or upper thorax with the sheet.

**Figure 3:**
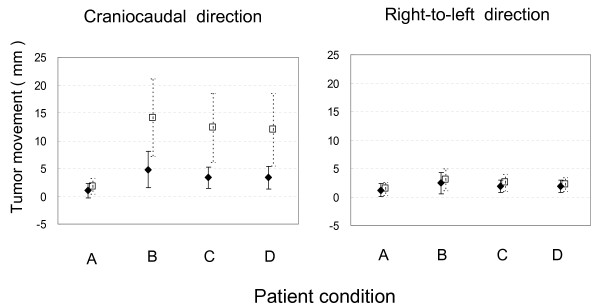
**Amplitude of tumor movement under conditions A, B, C and D in the craniocaudal and right-to-left directions in the upper and lower lung fields**. Black diamond: tumors in the upper lung field; open square: tumors in the lower lung field. A: breath holding; B: free breathing; C: free breathing covering the patient's lower body up to the abdomen with a clear plastic sheet; D: free breathing covering the patient's lower body up to the thorax with a clear plastic sheet. Bars represent SD.

We defined the upper lobe in the right lung and the segment 1+2 and 3 in the left lung as the upper lung field, and the rest of the lung as the lower lung field. The tumor movement in the lower lung field was much greater than in the upper lung field in the CC direction (Table 4 and Figure 3). In both lung fields, respiratory tumor movements were significantly reduced under conditions C and D compared with the free-breathing condition B in both CC and RL directions (Table 5). Again, however, there was no difference between conditions C and D in both fields. Under conditions C and D, decrease of tumor movement ≥ 3 mm in the CC direction was observed in 38% of the patients in the lower lung field, whereas it was seen in 17% in the upper lung field.

**Table 4:** Amplitude of tumor movement in the upper and the lower lung field (mm)

	Craniocaudal direction				Right-to-left direction			
	Upper lung field (n = 29)				Upper lung field (n = 29)			
	A	B	C	D	A	B	C	D
		
Range	0 – 5	1 – 15	0 – 8	0 – 8	0 – 5	1 – 10	0 – 5	0 – 5
Mean ± SD	1.0 ± 1.3	4.8 ± 3.3	3.3 ± 1.9	3.3 ± 2.0	1.2 ± 1.1	2.4 ± 1.8	1.9 ± 1.1	1.9 ± 1.1
								
	Lower lung field (n = 26)				Lower lung field (n = 26)			
	A	B	C	D	A	B	C	D
		
Range	0 – 5	2 – 25	1 – 24	1 – 25	0 – 5	0 – 8	0 – 5	0 – 5
Mean ± SD	1.8 ± 1.4	14.2 ± 6.9	12.4 ± 6.3	12.0 ± 6.5	1.5 ± 1.1	3.1 ± 1.9	2.5 ± 1.6	2.2 ± 1.2

**Table 5:** Reduction of tumor movement in the upper and the lower lung field

	Craniocaudal direction			Right-to-left direction		
	Upper lung field			Upper lung field		
	B – C†	B – D†	C – D†	B – C†	B – D†	C – D†
		
≤ - 3 mm	0	0	1	0	0	0
- 2 ≤, ≤ 2 mm	24	24	28	27	27	29
≥ 3 mm	5	5	0	2	2	0
p*	0.0044	0.0046	-	0.024	0.029	0.71
						
	Lower lung field			Lower lung field		
	B – C†	B – D†	C – D†	B – C†	B – D†	C – D†
		
≤ - 3 mm	2	2	3	0	0	0
- 2 ≤, ≤ 2 mm	14	14	19	24	22	26
≥ 3 mm	10	10	4	2	4	0
p*	0.032	0.0071	0.48	0.016	0.0051	0.043

p*: Paired *t*-test for mean values of tumor movement.

X – Y†: Amplitude of tumor movement in condition X minus that in condition Y.

Relationship between pulmonary function and tumor movement was analyzed. Thirteen and 10 tumors in the upper and lower lung fields, respectively, were in patients with normal pulmonary function. Ten, 2 and 4 tumors each in the upper and lower lung fields were in patients with obstructive dysfunction, those with constrictive dysfunction and those with mixed dysfunction, respectively. The correlation coefficients between %VC and FEV 1.0% and amplitudes of tumor movement in the CC and RL directions were calculated, but there was no significant correlation between respiratory function and the amplitude of tumor movement in both directions (data not shown).

### Percutaneous Oxygen Saturation Level

**Figure 4:**
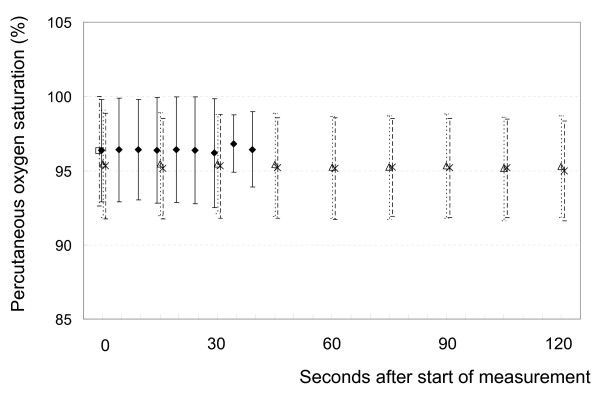
**SpO_2 _change under conditions A (black diamond), B (open square), C (open triangle) and D (X)**. A: breath holding; B: free breathing; C: free breathing covering the patient's lower body up to the abdomen with a clear plastic sheet; D: free breathing covering the patient's lower body up to the thorax with a clear plastic sheet. Bars represent SD.

Changes in SpO_2 _levels are shown in Figure 4. There were 10 cases in which SpO_2 _decreased by 3% or more; in 1 case, the decrease was as large as 10%. There was 1 case in which SpO_2 _increased by 3% or more under the breath-holding condition A. On average, the SpO_2 _level did not decrease under condition A. There were 5 cases in which SpO_2 _decreased by 3% or more and 4 cases in which SpO_2 _increased by 3% or more under both conditions C and D. The average SpO_2 _level did not decrease under both conditions C and D. The change of SpO_2 _was not different between conditions C and D (*p *= 0.56).

### Clinical Outcomes

In actual treatment, we used the patient condition C, i.e., free breathing covering the patient's lower body up to the abdomen with a clear plastic sheet. The mean ± SD of PTV volumes was 53 ± 30 cm^3^, with a range of 8.1 to 146 cm^3^.

The median follow-up period was 32 months (range, 24 to 52 months). For follow-up after the SBRT, CT examination was performed at 2-month intervals until the 6th months, and every 2 to 4 months thereafter. FDG-PET was performed whenever necessary. Local recurrence was suspected by enlargement of a fibrotic mass on CT images without sign of inflammation, and diagnosed by high uptake on FDG-PET and/or biopsy.

The local control rate was 80% for all lesions at 3 years. In 42 primary NSCLC treated, 30 lesions were stage IA and 12 were stage IB. We had no patient treated with 44 Gy in 4 fractions. All stage IA lesions were treated with 48 Gy in 4 fractions, and stage IB lesions were treated with 52 Gy in 4 fractions. Local recurrence developed in 8 (5 among stage IA patients and 3 among stage IB). Regional lymph nodes recurrence occurred in 6 (3 among stage IA and 3 among stage IB). Distant metastasis appeared in 13 patients (8 among stage IA and 5 among stage IB). There was no difference in regional and systemic progression between patients with and without FDG-PET staging. At 3 years, overall survival was 76%, cause-specific survival was 92%, progression-free survival was 54%, and local progression-free survival was 76%. For stage IA, they were 83%, 96%, 55% and 79%, respectively. For stage IB, they were 58%, 82%, 55% and 74%, respectively.

Toxicity was evaluated using the Common Terminology Criteria for Adverse Events Version 3. Grade 2 radiation pneumonitis (symptomatic but not interfering with activity of daily life) was observed in 7 patients. At 3 years, the cumulative incidence was 17%. The rate for stage IA and IB was 17% and 19%, respectively. Other adverse events were grade 1 atelectasis seen in 3 patients, pleural effusion of grade 1 and 2 in 5 and 1, respectively, grade 2 esophagitis in 2, grade 1 dermatitis in 2, grade 2 rib fracture in 1, and soft-tissue swelling in 2.

## Discussion

There are many reports on the respiratory tumor movement using fluoroscopy, portal image, CT, and MRI [5,6,8-15]. The correlation between tumor location and amplitude of movement was analyzed in several studies [8,9,11-13,15]. Onimaru et al. [16] evaluated the amplitude of the tumor motion and the difference in the amplitude according to the marker sites on a plain chest X-ray film. Our results were comparable to theirs. A limitation of our study was that motion in the AP direction was not measured. Movement in the AP direction often could not be seen well with fluoroscopy because tumor overlapped mediastinal structures [8]. Tumor movement in the AP direction is much less than that in the CC direction, but on CT images taken at 3 phases, it was well recognized and the range of movement was included in the PTV. In future studies, 4-dimensional management of tumor movement should be warranted.

To irradiate the tumor precisely and to decrease the irradiated volume of the normal lung, various methods have been developed. They can be classified into 5 major categories; motion-encompassing method, respiratory-gating method, breath-hold method, forced shallow-breathing with abdominal compression method, and real-time tumor-tracking method [17]. In any method, accurate setup is necessary. Repositioning accuracy of some commercially available immobilization devices was reported to be acceptable [6,7,18,19]. They also have an instrument for reducing the tumor movement. Negoro et al. [6] reported the effectiveness of the stereotactic body frame. The average tumor movement in the CC direction during free respiration was 7.7 mm, with a range of 0 to 20 mm. In the patients in whom tumor movement was greater than 5 mm, the abdominal press reduced the tumor movement significantly from a range of 8 to 20 mm to a range of 2 to 11 mm (*p *= 0.0002). Our method is a combination of a motion-encompassing method and a forced shallow-breathing method with abdominal compression. Using the BodyFIX system, the tumor movements were modestly but significantly reduced compared with free-breathing status. However, reduction of tumor movement by 3 mm or more in the CC direction was obtained in only 27% of the patients, and in the rest of the patients, suppression of tumor movement was not considered to be satisfactory. Therefore, the use of the BodyFix system appeared to have limited influence on the ITV size. Covering the patient's lower body up to the abdomen and up to the thorax with a clear plastic sheet showed no differences. So, in practical SBRT, we covered the body up to the upper abdomen. From these considerations, the major purpose of using this system appeared to be to position the patient accurately and the second one was to suppress tumor movement modestly without influencing oxygenation status of tumors. Other strategies may be necessary in cases with large tumor movement.

Regarding other uncertainties associated with SBRT, patient positioning and especially base-line shifts of the target position have been reported to be the most relevant uncertainties [20,21]. To decrease these uncertainties, we used verification CT before the first and third fractions of SBRT and AP and lateral portal images every time. These verification methods may be less accurate than the currently available image-guidance techniques like cone beam CT. Such newer image guidance systems are desirable in future SBRT. Regarding intrafractional motions, we did not measure them, but the BodtFix system should be useful in reducing this uncertainty.

We monitored the change of SpO_2 _under conditions A, C and D, because we had a concern that SpO_2 _might decrease by suppression of respiration especially in patients with low pulmonary function. If SpO_2 _decreases significantly, tumor response might become poorer due to the increase in hypoxia [22]. There was 1 case in which SpO_2 _decreased by 10% under breath-holding condition A. It did not decrease under conditions C and D in the same patient. In a few patients, SpO_2 _decreased by 3% or 4% at maximum under conditions C and D, but in the other patients, SpO_2 _did not decrease at all while using the BodyFIX system. Therefore, it was concluded that the BodyFIX system does not significantly influence SpO_2 _levels in the majority of patients.

Clinical outcome of our patients in terms of antitumor effect and toxicity compares favorably with that published recently [3,23], suggesting that our fractionation schedules are, at least, not inferior to those used by other investigators. The relatively low toxicity may not mainly result from the use of the BodyFix system. Follow-up periods are still short in a considerable proportion of patients, and we will continue further follow-up.

## Conclusion

Respiratory tumor movement was modestly suppressed by using the BodyFIX system, while the SpO_2 _level did not decrease. Although the system did not seem to be very useful to decrease the ITV, it appeared to be a simple and effective method for SBRT of lung tumors. Our method of SBRT was safe and the preliminary result was favorable.

## Competing interests

The authors declare that they have no competing interests.

## Authors' contributions

FB carried out the study and drafted the manuscript. YS indicated the design of the study and gave final approval of the version to be published. NT participated in analysis and interpretation of data. CIH, KO and SA participated in acquisition and analysis of data. HO participated in the design of the study and helped to perform the statistical analysis. CS participated in analysis and interpretation of data and helped to draft the manuscript. All authors read and approved the final manuscript.
